# Altered ciliary morphology reduces mechanosensation in a cystic kidney model as indicated by a mathematical model

**DOI:** 10.1038/s41598-026-39179-y

**Published:** 2026-03-01

**Authors:** Kanako Kumamoto, Hiroyuki Kagami, Sei Saitoh, Shiori Yamada, Mami Matsumoto, Nobuhiko Ohno

**Affiliations:** 1 Advanced Medical Research Center for Animal Models of Human Disease, Fujita Health University, 1-98 Dengakugakubo, Kutsukake-cho, Toyoake, Aichi 470-1192 Japan; 2 Graduate School of Nursing, Nagoya City University, 1, Kawasumi, Mizuho-cho, Mizuho-ku, Nagoya, Aichi 467-8601 Japan; 3 Department of Disease Systems Analysis Medicine, Fujita Health University Graduate School of Medical Science, Toyoake, Aichi Japan; 4 Yamaguchi Ladies Clinic, 2-7-1, Kakeage, Minami-ku, Nagoya-shi, Aichi 457-0007 Japan; 5 Section of Electron Microscopy, Supportive Center for Brain Research, National Institute for Physiological Sciences, Okazaki, 444-8787 Japan; 6 Department of Developmental and Regenerative Neurobiology Institute of Brain Science, Nagoya City University Graduate School of Medical Sciences, Nagoya, 467-8601 Japan; 7 Division of Ultrastructural Research, National Institute of Physiological Sciences, 38 Saigonaka, Meidaiji-cho, Okazaki, Aichi 444-8585 Japan; 8 Department of Anatomy, Division of Histology and Cell Biology, Jichi Medical University, 3311-1 Yakushiji, Shimotsuke, Tochigi 329-0498 Japan; 9 Present Address: Department of Advanced Diagnostic System Development, Graduate School of Medical Sciences, Fujita Health University, Toyoake, Japan

**Keywords:** Biophysics, Computational biology and bioinformatics, Engineering, Physics

## Abstract

This study investigates the biomechanical properties of primary cilia in healthy kidneys and an early-stage cystic kidney model (CKM), focusing on their role in flow-mediated mechanosensation. Morphological analysis showed that CKM cilia are longer, more curved, and exhibit disrupted axonemal integrity compared with normal cilia. To evaluate the effect of such structural changes on bending and stiffness, which may affect the drag force, shear stress and PC1/PC2 complex activation, we developed a mathematical model simulating urine-flow-induced drag. The model predicts that longer and curved cilia experience only one-fourth of the drag force of shorter and straight cilia under identical flow conditions. Remarkably, addition of 5% glucose to drinking water, which was reported to increase water intake, was predicted by the model to elevate urine flow to levels sufficient to partially normalize ciliary length and tubular morphology in CKM kidneys. These findings indicate that ciliary deformation impairs mechanosensation, contributing to cystogenesis, and that restoring mechanical stimulation may mitigate disease progression. Beyond estimating the urine volume required for therapeutic effect, the model offers a framework for developing interventions targeting ciliary mechanotransduction, which could be particularly useful when fluid-loading strategies are not feasible. This approach highlights the potential of combining morphological analysis, biophysical modeling, and mechanobiology to better understand and treat early cystic kidney disease.

**Supplementary Information:**

The online version contains supplementary material available at 10.1038/s41598-026-39179-y.

## Introduction

Cells can sense and respond to their environment by converting physical stimulation into biochemical signals, a process known as mechanotransduction^1,2^. A variety of force-sensing mechanisms have been reported in cells, including integrin-mediated adhesions^3^, stretch-sensitive channels^4,5^, and fluid-flow sensors^6^.

Primary cilia act as efficient mechanosensory organelles for fluid flow owing to their structural characteristics^7^. Bending a primary cilium by fluid flow^8^, optical tweezers^9^, or micropipettes^10^ initiates intracellular calcium release, indicating that the primary cilium functions as a physiological flow sensor. However, the biological significance of this function remains unclear, partly due to an incomplete understanding of the cilium’s mechanical properties within tissues under fluid-flow conditions^11–13^. Nevertheless, the quantification and modeling of ciliary mechanical properties have a long history. Detailed analyses of ciliary deformation in vivo, combined with mathematical modeling, have advanced our understanding of how these properties enable cilia to decode mechanical signals^14–16^. Primary cilia are composed of nine microtubule doublets, each containing a complete A-tubule (~ 13 protofilaments) and a semicircular B-tubule (~ 10 protofilaments)^17^. A-tubules are enriched in α-tubulin acetylation, whereas B-tubules harbor higher levels of polyglutamylation^18,19^. These post-translational modifications contribute to the stabilization of axonemal microtubules and the regulation of ciliary function, including intraflagellar transport and motility^18,20^.

This study aims to develop a mathematical model to examine how morphological alterations of primary cilia in cystic kidney disease may reduce the drag force and shear stress experienced at the ciliary surface. (Drag force per unit area: Under conditions characterized by low Reynolds numbers, dominant viscous forces, and slender geometry, the drag force acting on the cilium is primarily attributable to shear stress along its surface. Accordingly, the drag per unit length can be approximated based on the local shear stress.) Specifically, the model evaluates the urine flow rate required for cilia in cystic kidneys to experience the same level of shear stress as those in wild-type kidneys. Morphological abnormalities such as elongation and curvature are expected to decrease hydrodynamic drag, which could decrease the function of mechanosensory complexes possibly including polycystin-1 (PC1) and polycystin-2 (PC2), which regulate calcium flux in response to ciliary bending^21–23^. Because calcium signaling through PC1/PC2 is closely associated with cystogenesis, determining the compensatory urine flow necessary to restore shear stress is of both biological and clinical importance.

Urine flow in the renal tubule is tightly regulated by arginine vasopressin (AVP), which activates the V₂ receptor in collecting duct principal cells, elevating intracellular cAMP levels. This increase promotes aquaporin-2 (AQP2) transcription and apical membrane insertion, enhancing water reabsorption and reducing urine output^24^. In polycystic kidney disease, suppression of the AVP–cAMP–AQP2 axis is considered beneficial, as it increases urine flow and may slow cyst growth. High-water intake (HWI) lowers plasma AVP and renal cAMP levels, thereby reducing AQP2 expression and increasing urine output^25,26^. In experimental models, suppression of AQP2 has been shown to delay cyst progression^27^. Hopp et al. also proposed that HWI-induced vasopressin depletion itself may contribute to cyst suppression^28^. Because it is difficult in vivo to separate the effects of increased urine flow from those of reduced AVP or cAMP levels, a mathematical model provides a valuable tool to isolate the mechanical contribution of urine flow alone to ciliary function.

We constructed a mathematical model using rats with either normal kidneys or cystic kidney models (CKMs), characterized by tortuous primary cilia and dilated renal tubules, to predict the distribution of drag force on the cilia. The drag force per unit area (shear stress) exerted on the primary cilia of epithelial cells in both normal and cystic kidneys was compared. The analysis revealed that, to achieve an equivalent drag force per unit area, the urine flow rate in cystic kidneys needed to be approximately 3.49 times higher than that in wild-type kidneys. Consistently, partial normalization of cilia length, structure, and tubular diameter was observed in CKMs by addition of 5% glucose to drinking water, which was reported to increase the water intake and urine flow. These findings suggest that impaired mechanosensation resulting from altered ciliary morphology could be mitigated by restoring shear stress through increased urine flow. Moreover, this mathematical model provides a useful tool for estimating the urine volume required for therapeutic benefit and offers a conceptual framework for developing novel treatments targeting ciliary mechanotransduction—particularly valuable when fluid-loading strategies are not clinically feasible.

## Results

### Morphology of primary cilia in the WT and cystic kidney models (CKM)

**Fig. 1:**
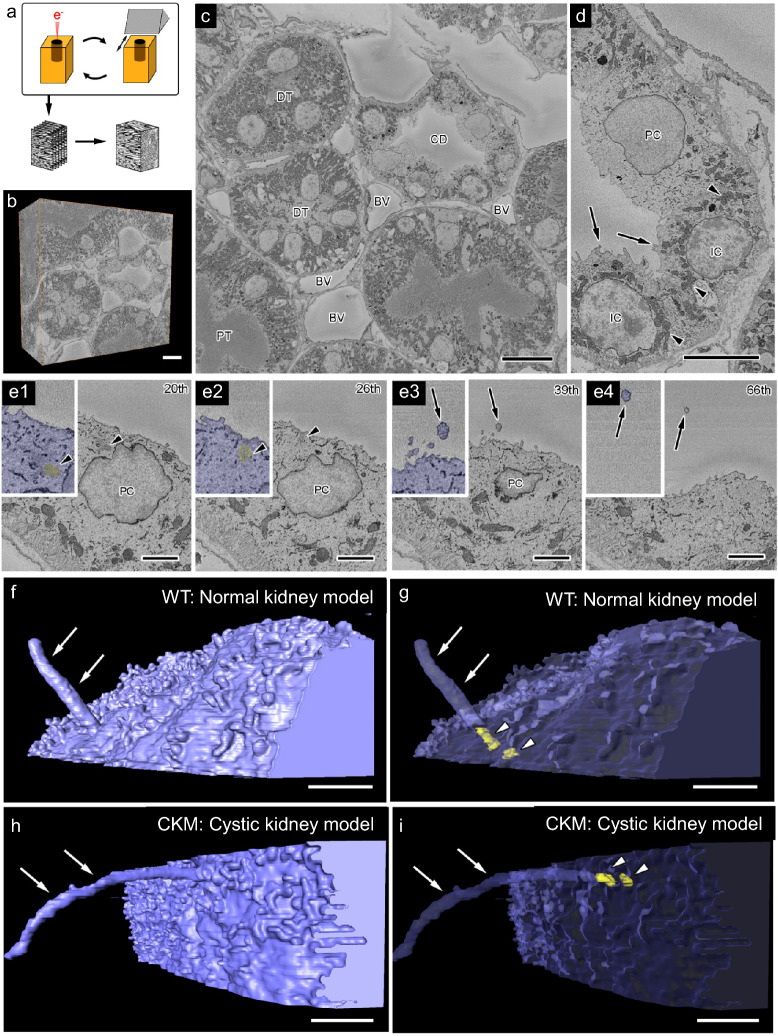
**Three-dimensional (3D) reconstruction of a primary cilium in collecting duct (CD) of the kidney.** Definition of primary cilium length and tortuosity is illustrated schematically based on the 3D reconstructions, showing the curved length (arc length, **a**) measured along the ciliary centerline and the straight-line distance between the ciliary base and tip (chord length, **b**). Ciliary tortuosity was defined as the ratio of arc length to chord length (**a**/**b**), where a value of 1 indicates a perfectly straight cilium in three-dimensional space. In SBF-SEM, serial electron microscopic images are acquired by repeating scanning and slicing procedures, and 3D reconstruction is performed with the serial images (**a**). The reconstructed volume of rat kidney tissues is shown in (**b**). CDs are identified among proximal (PT) and distal (DT) tubules and blood vessels (BV) (**c**). In CD (**d**), principal cells (PC) with primary cilia are observed among intercalated cells (IC) with abundant mitochondria (**d**, arrowheads) and many luminal microfolds (d, arrows). In the serial images of PC (e1-4, blue), a basal body (e1, e2, arrowheads, yellow) and a primary cilium extended from the basal body (e3, e4, arrows) can be identified, and reconstructed (**f**-**i**, white arrows, white arrowheads). Cilia from WT (Normal kidney model: f, g and Supplementary Movie 1) and CKM (Cystic kidney model: h, i and Supplementary Movie 2) rats were reconstructed. The slice numbers are described in upper right corners (e1-4). Bars: 10 μm (**b**), 5 μm (**c**, **d**), 2 μm (**e**-**i**).

**Fig. 2:**
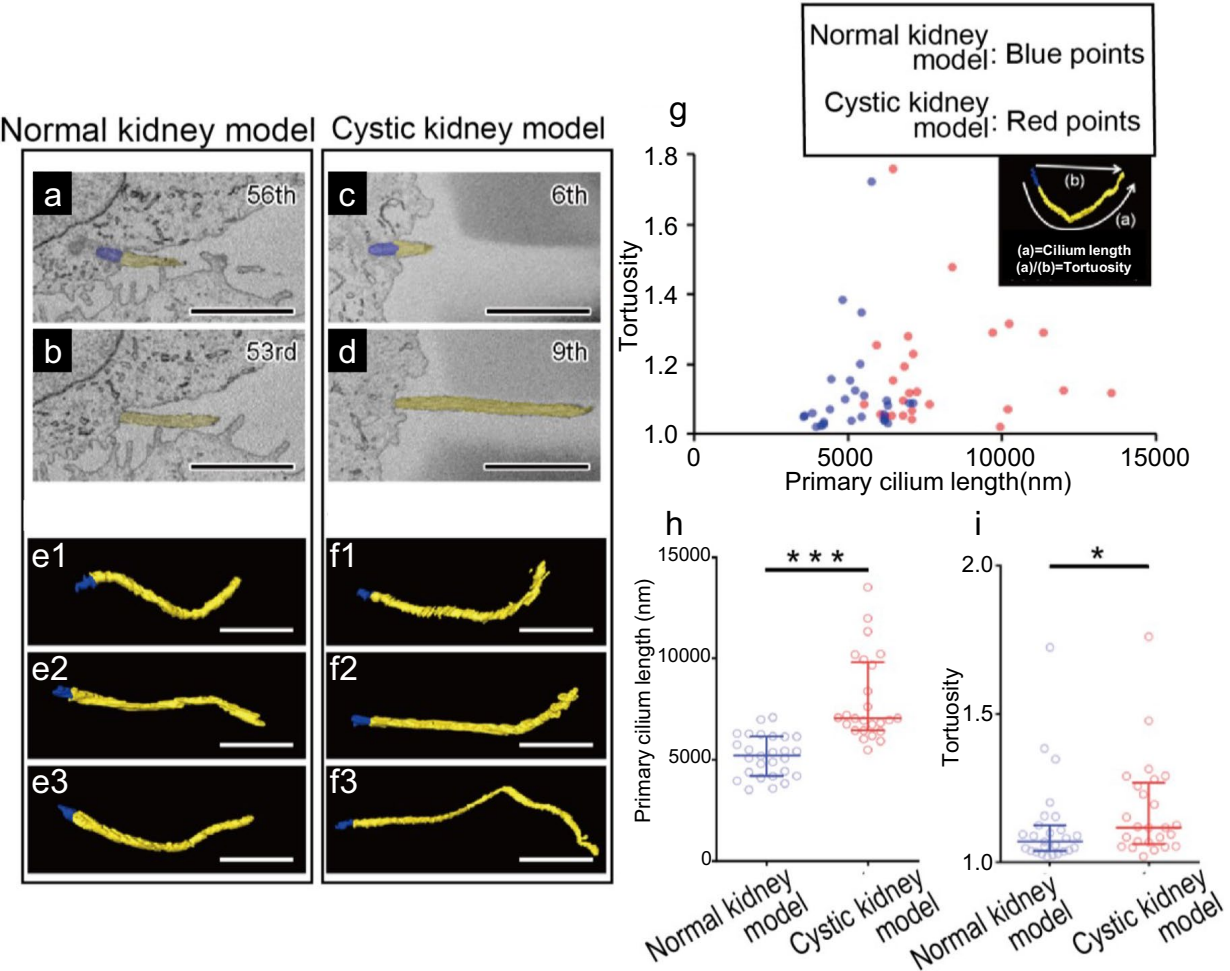
**Morphology of primary cilia in the Normal kidney model and Cystic kidney model (CKM).** Detailed observations reveal longer and more tortuous primary cilia in the principal cells of the outer medullary collecting duct in CKMs. Basal bodies (**a**,** c**; blue) and primary cilia (**a**–**d**; yellow) of principal cells in the outer medullary collecting duct are colored in the serial block-face scanning electron micrographs of Normal kidney model (**a**,** b**) and CKM (**c**,** d**) rats. The slice numbers are described in the upper right corners of the images. Representative reconstructions of basal bodies in the cytoplasm (**e**,** f**; blue) and extended primary cilia (**e**,** f**; yellow) of principal cells in Normal kidney model (e1-3) and CKM (f1-3) cells. In Fig. 2, the numbers of datasets and animals are 3 and 2, respectively, for both WT and the CKM model, and the number of analyzed cilia is n = 27 for WT and n = 25 for the CKM model. Primary cilium length was measured as arc length along the curved centerline. Chord length (straight-line distance between base and tip) was also extracted from the same 3D reconstructions. The scatter plot (**g**) and graphs (**h**,** i**) show ciliary length (**g**,** h**) and tortuosity (**g**,** i**) of primary cilia measured in Normal kidney model and CKM. The mean ± SD (panel h) or median with interquartile ranges (panel i) are presented. Statistical significance is indicated as *** *p* < 0.001 (t-test: panel h) and * *p* < 0.05 (Mann–Whitney U test: panel i). Scale bar: 2 μm.

Because the genes responsible for cystic kidney disease, including PC1, PC2, Nek8, and Pkhd1, are localized to primary cilia, ciliary function likely plays an important role in the mechanical sensing involved in renal tubule dilation. The estimation of drag force acting on primary cilia in primitive urine is thought to depend on their length and morphology. Therefore, as a first step, we observed the length and tortuosity of primary cilia in the principal cells of the outer medullary collecting duct in both WT and CKM rats using serial block-face scanning electron microscopy (SBF-SEM) (Fig. 1, 2). In SBF-SEM, serial electron microscopic images are acquired by repeating scanning and slicing procedures, and three-dimensional reconstructions can be generated from these serial images (Fig. 1a, b). Three-dimensional reconstructions were generated from collecting duct samples obtained from two independent CKM rats. Collecting ducts were identified among proximal and distal tubules as well as blood vessels (Fig. 1c). Within the collecting ducts, principal cells bearing primary cilia were distinguished from intercalated cells, which contain abundant mitochondria and numerous luminal microfolds (Fig. 1d).

In serial images of principal cells (Fig. 1e1–e4, blue), primary cilia (Fig. 1e3, e4, arrows) extending from basal bodies (Fig1e1, e2, arrowheads, yellow) could be identified and reconstructed (Fig. 1f–i). This approach enabled us to reconstruct and compare the entire length and trajectory of cilia from principal cells in both WT (Fig. 1f, g; Supplementary Movie1) and CKM (Fig1h, i; Supplementary Movie2) rats. By segmenting the cilia from basal bodies (Fig. 2a, c) to their tips (Fig. 2b, d) and reconstructing multiple cilia in three dimensions (Fig. 2e1–e3, f1–f3) from WT (Fig. 2a, b, e1–e3) and CKM (Fig. 2c, d, f1–f3) kidneys, we found that primary cilia in the CKM were longer and more tortuous than those in the WT (F2e1–e3, f1–f3).

Next, we performed a quantitative analysis comparing ciliary length and tortuosity (Fig. 2g–i). The median ciliary length in the CKM was 7,051 nm, markedly greater than that in the WT model (5,203 nm) (Fig. 2g, h). Furthermore, the median tortuosity of CKM cilia was 1.176, which was significantly higher (P < 0.05) than that in WT cilia (1.070) (Fig. 2g, i). Primary cilia tortuosity was quantified in collecting ducts from CKM and WT rats. Tortuosity values were broadly distributed in both groups; however, CKM cilia exhibited a statistically significant increase in central tendency (median ± IQR: CKM = 1.117 ± 0.188 vs WT = 1.070 ± 0.077; Mann–Whitney U test, *p* = 0.041). Moreover, 48% of CKM cilia exceeded the 75th percentile of the WT distribution, indicating that the elevated tortuosity represents a population-level shift rather than being driven solely by a small subpopulation. Although a bi-modal pattern cannot be strictly excluded, our dataset does not provide statistical evidence supporting a separable sub-population. Rather, the distributions of tortuosity in WT and CKM show substantial overlap, with an enrichment of higher-curvature profiles in the CKM condition, indicating a shift in the distribution toward increased bending rather than a categorical distinction between groups (Fig. 2g, i).

### Altered morphology of primary cilia in the CKM: elongation and bending due to distinct internal structures compared to the wild type

**Fig. 3:**
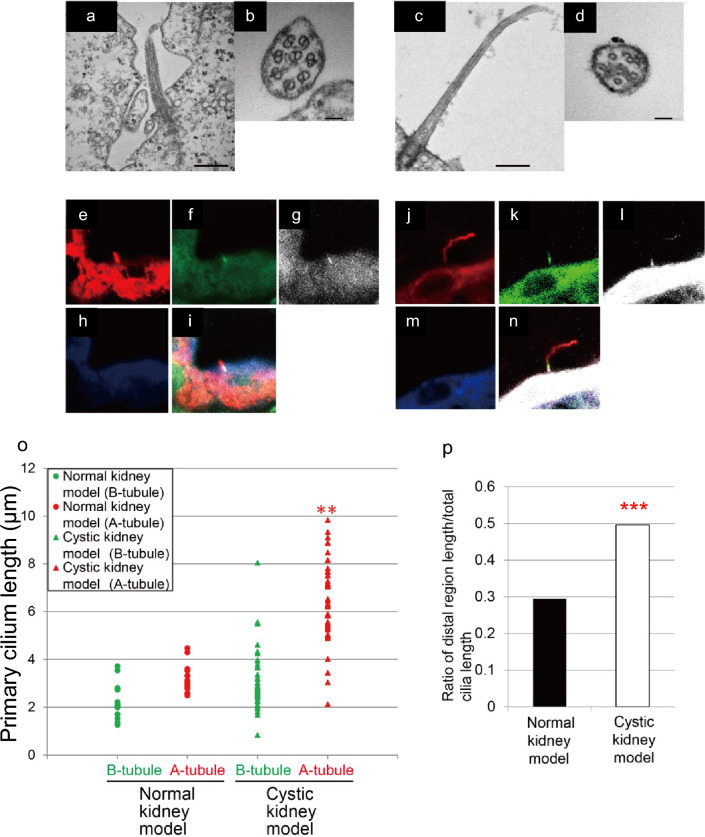
**Internal structure of primary cilia with a long bent in the cystic kidney model (CKM).** Ultrastructural analysis of the primary cilium of principal cells in the collecting duct of the Normal kidney model and CKMs. Representative TEM images of a longitudinally sectioned primary cilium of Normal kidney model (**a**) and a CKM (**c**). Attention is drawn to the extreme length and curvature of the cilia in the CKM (**c**). Enlargements of representative cross-sections of the primary cilium of WT (**b**) and the CKM (**d**). TEM images of cross-sections of a primary cilium at the distal region in WT show reduction the nine to eight axoMT but still making microtubule doublets (**b**). Almost all axonemes were microtubule singlets, despite the middle part of the primary cilia (**d**). Scale bars are 500 nm (**a**,** c**) and 50 nm (**b**,** d**). The axoneme comprises doublets built by A-tubule (red: acetylated tubulin; e and j) and glutamylated B-tubule [green: ≥ 3 (**f **and** k**) or white ≥ 1 (**g** and** l**) glutamate residue] at the proximal part of cilia^32,73^. The doublets transit to the non-glutamylated A-tubule singlet at the distal parts of the cilia. Attention is drawn to the extreme distal length and curvature of the cilia in the CKM (**n**). The doublets transit to the non-glutamylated A-tubule singlet, in the distal part of the cilia (red; acetylated tubulin). Glutamylated B-tubule is proximal (green; ≧3 glutamate residues). In the CKM, primary cilia displayed a notable hyperextension in the distal portions of their axonemes compared to their proximal parts. This morphological feature was predominantly observed in tubules with an increased diameter. Internal structure (**o**) and ratio of distal part (**p**) in the primary cilia of Normal kidney model and model rat renal collecting ducts. The lengths of the A- and B-tubules within the microtubule doublets of primary cilia in both Normal kidney model and CKMs were measured. Subsequently, the ratio of the distal length (difference between A- and B-tubule lengths) to the total A-tubule length was calculated. Primary cilium length represents the calculated straight-line distance (chord length) between the ciliary base and tip derived from orthogonal-axis measurements in Fiji. In Fig. 3, the number of animals was N = 3 for WT and N = 3 for the CKM model, and the number of analyzed cilia was n = 18 for WT and n = 48 for the CKM model. Data are presented as mean ± SD. Statistical analysis was performed using one-way ANOVA followed by Tukey’s post hoc test, with **p < 0.01 (panel o) and ***p < 0.001(Mann–Whitney U test: panel p) considered statistically significant.

To elucidate the mechanisms underlying primary cilium elongation and bending in the CKM, we analyzed the axonemal structure at the distal region. The axoneme, a core component of primary cilia, typically comprises nine microtubule doublets. These structures were examined in both WT and CKM kidneys (Fig. 3a–d). In the WT kidney, axonemes in the distal region exhibited a reduction in the number of microtubules from nine to eight while maintaining the characteristic doublet structure (Fig. 3a, b). In contrast, CKM axonemes displayed a distinct configuration, consisting primarily of microtubule singlets (Fig. 3c, d).

To determine which elements of the microtubule doublet (A- and B-tubules) contribute to ciliary elongation, we stained A- and B-tubules with an acetylated tubulin antibody to visualize their full length and stained B-tubules with antibodies recognizing ≥ mono- or ≥ tetra-glutamylated tubulin. The samples were then analyzed using confocal microscopy (Fig. 3e–n). The total length of microtubules in primary cilia from both WT and CKM kidneys was strongly labeled with acetylated tubulin from the base to the tip (WT, red: Fig. 3e, i; CKM, red: Fig. 3j, n). Regions corresponding to B-tubules, stained with antibodies against ≥ 1 or ≥ 4 glutamyl residues, were strongly labeled approximately 2 µm from the base (WT: ≥ 1 glutamate, Fig. 3g; ≥ 4 glutamates, Fig. 3f; CKM: ≥ 1 glutamate, Fig. 3l; ≥ 4 glutamates, Fig. 3k).

From these data, the distal segment (DS) length—composed of singlet microtubule structures—was calculated as the difference between the entire ciliary length (acetylated tubulin-stained region) and the length of the B-tubule-stained proximal segment (PS) (Fig. 3i, n; the red tip region represents the DS). Interestingly, in WT kidneys, the DS was short (average total length = 3 µm; average DS length = 0.9 µm), whereas in CKM kidneys, the DS accounted for more than 50% of the cilium (average total length = 6 µm; average DS length = 3 µm) (Fig. 3o, p).

Thus, in CKMs, more than half of the primary ciliary internal structure may consist of elongated singlet microtubules, which are less rigid than the doublets found in WT kidneys. These structural changes, including elongation and increased curvature, are expected to alter the interaction between primary cilia and luminal flow, motivating a quantitative biomechanical analysis. Moreover, decreased stiffness and increased curvature are likely to redistribute drag-induced bending moments along the ciliary shaft, diminishing the transmission of shear stress to the ciliary base, where mechanosensory signaling is believed to be initiated.

To quantitatively assess these biomechanical effects and evaluate the potential for flow-mediated compensation, we developed a mathematical model to simulate the drag forces acting on primary cilia with varying morphologies.

Our intention in developing the mathematical model was not to reproduce the full morphological complexity observed by electron microscopy, but to isolate and compare the mechanical consequences of two idealized configurations—short and straight versus long and bent—motivated by statistically supported trends in length and tortuosity.

### Mathematical model of the force acting on the short and straight primary cilia.

The force acting on the primary cilia in the tubule is mathematically estimated below.

The drag $D$ to an object in a fluid can be written as:

1 $$D=\frac{1}{2}\rho {V}^{2}S{C}_{D}$$

where $\rho$ denotes the density of the fluid, $V$ is the relative velocity between the object and fluid, $S$ is the area of the object perpendicular to the flow direction, and ${C}_{D}$ is the drag coefficient. Assuming that the Reynolds number (*Re*) is $<2$ because the flow in the tubule is sufficiently slow, ${C}_{D}$ can simply be expressed as follows:

2 $${C}_{D}=\frac{24}{Re}$$

$Re$ can be written as:

3 $$Re=\frac{\rho VL}{\mu }$$

where $L$ denotes the characteristic length, and $\mu$ is the viscosity coefficient. From the above, $D$ can be written as:

4 $$D=\frac{12\mu SV}{L}$$

First, we consider a case in which the primary cilia extend vertically from the tubular wall.

Assuming that the diameter of the circle of the cross section of the primary cilia is $a$, the drag $D\left(r\right)$ acting on the portion of the length of $dr$ at position $r$ in the vertical direction from the tubular wall can be written as follows:

5 $$D\left(r\right)=\frac{12\mu aV\left(r\right)}{L}dr$$

Here, since $V$ is a function of $r$, it is written as $V\left(r\right)$.

We modeled the renal tubule as a cylindrical tube with a diameter of $R$, containing a fluid exhibiting the laminar flow characteristics of a viscous fluid. Orienting the $x$-axis along the direction of flow and the $r$-axis perpendicular to it, with the tubule wall located at $r=0$, the $V\left(r\right)$ is at $0\le r\le \frac{R}{2}$ as follows:

6 $$V\left(r\right)=-\frac{1}{4\mu }\frac{dp}{dx}{r}^{2}$$

where $p$ denotes the fluid pressure. For simplicity, assuming Eq. (7), Eq. (8) can be obtained.

7 $$-\frac{dp}{dx}=\alpha \left(constant\right)$$

8 $$D\left(r\right)=\frac{3a\alpha {r}^{2}}{L}dr$$

From the above, force $D$ acting on the primary cilia of length $l$ can be estimated as follows:

9 $$D={\int }_{0}^{l}\frac{3a\alpha {r}^{2}}{L}dr=\frac{a\alpha {l}^{3}}{L}$$

## Mathematical model of the force acting on the long and bent primary cilia.

**Fig. 4:**
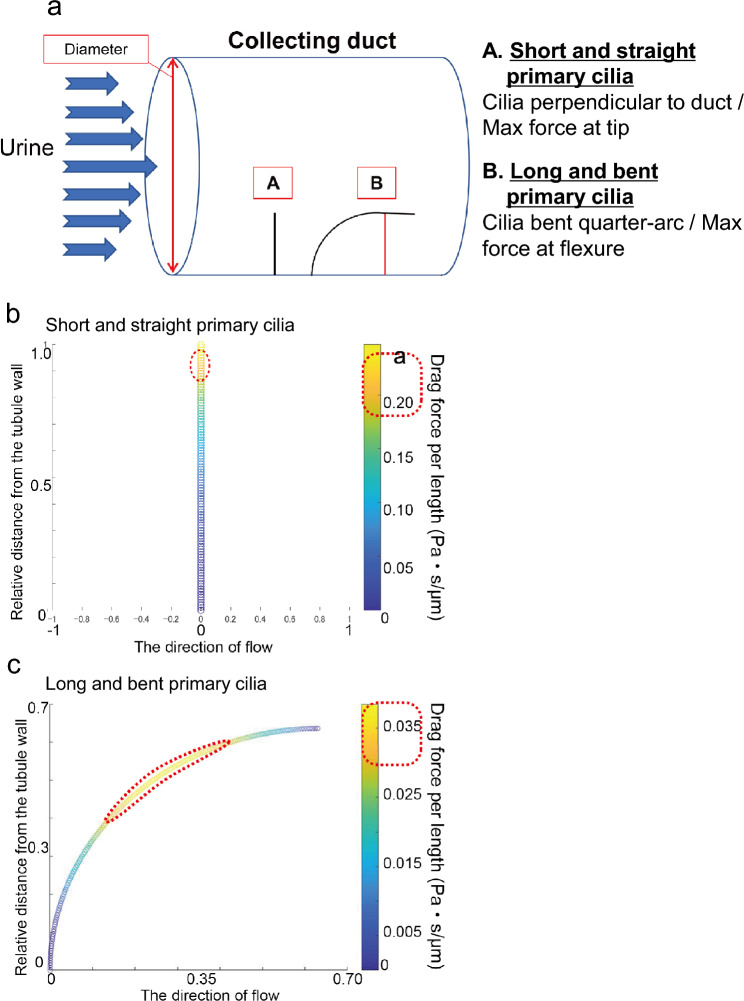
**Mathematical model of the force acting on the primary cilia in kidney renal tubules.** (**a**) A schematic model of the short and straight (**A**) and long and bent (**B**) primary cilia. Blue arrows on the left indicate urine flow. The blue cylinder represents the collecting duct. The model of short and straight cilia is a cantilever shape of primary cilia perpendicular to the collecting duct wall (**A**). In the model of long and bent cilia, primary cilia are represented as curved, forming approximately a quarter-circle arc (**B**) , used as an idealized, analytically tractable shape motivated by observed differences in ciliary length and curvature, rather than a direct reconstruction of ultrastructure. Drag force per length of bent and long cilium in each kidney model (the model of short and straight cilia: Fig. 4b; the model of long and bent cilia: Fig. 4c) These schematic models were calculated using the drag force per length using Eqs. (8: for the model of short and straight cilia) and (15: for the model of long and bent cilia) and plotted by MATLAB. The tips of the short and straight cilia are predicted by the model to experience the highest forces, indicating a distal concentration of mechanical loading (the model of short and straight cilia: Fig. 4b). In contrast, in the long and bent cilia, the highest force position was predicted to move from the tip to the flexure (CKM: Fig. 4c), reflecting a redistribution of force along the curved shaft.

Next, we considered the case in which the primary cilia extended from the tubular wall as bent cilia (Fig. 4 a-B). For simplicity, it was assumed that the primary cilia were bent in a quarter of an arc (Fig. 4a-B). We used a quarter-circle arc as an analytically tractable idealization of bent cilia because it provides a defined 90° tangent-flow alignment point and allows mechanistic comparison before extending to irregular geometries. With the point of contact between the primary cilia and tubular wall as the origin, the $r{\prime}$-axis (Fig. 4a-B: Red line) is taken along the primary cilia towards the tip of the primary cilia. The angle between the straight dotted line connecting the center of the circle and the location $r{\prime}$ of the primary cilia and the tubular wall is defined as $\theta$(Fig. 4a-B).

Because the area $S$ perpendicular to the direction of the flow of the object is expressed as in Eq. (10), taking the $r$-axis vertically upward from the tubular wall, the drag ${D}_{C}\left(r{\prime}\right)$ acting on the length of $dr{\prime}$ at the position of $r{\prime}$ can be written as in Eq. (11).

10 $$S=a\mathit{cos}\theta dr{\prime}$$

11 $${D}_{C}\left(r{\prime}\right)=\frac{12\mu a\mathit{cos}\theta V\left(r\right)}{L}dr{\prime}$$

Here, because the relations of Eqs. (12)–(14) hold, Eq. (11) can be expressed as Eq. (15).

12 $$r=\frac{2}{\pi }l\mathit{sin}\theta$$

13 $$dr{\prime}=\frac{2}{\pi }ld\theta$$

14 $$V\left(r\right)=\frac{\alpha }{4\mu }{r}^{2}$$

15 $${D}_{C}\left(\theta \right)=\frac{12\mu a\mathit{cos}\theta }{L}\frac{\alpha }{4\mu }{\left(\frac{2}{\pi }l\mathit{sin}\theta \right)}^{2}\frac{2}{\pi }ld\theta =\frac{24a\alpha {l}^{3}}{L{\pi }^{3}}\mathit{cos}\theta \left(1-{cos}^{2}\theta \right)d\theta$$

From the above, the force ${D}_{C}$ acting on the primary cilia of length $l$ curved in an arc can be estimated as follows:

16 $${D}_{C}=\frac{24a\alpha {l}^{3}}{L{\pi }^{3}}{\int }_{0}^{\frac{\pi }{2}}\mathit{cos}\theta \left(1-{cos}^{2}\theta \right)d\theta =\cdots ={\left(\frac{2}{\pi }\right)}^{3}\frac{a\alpha {l}^{3}}{L}={\left(\frac{2}{\pi }\right)}^{3}\sim 0.258\times D$$

That is, it is approximately $1/4$ of the force acting on the primary cilia of length $l$. This is exactly the same as the force acting on the primary cilia extending vertically from the tubular wall to the height of the radius of the circle but is different when considered per unit length.

**If the drag force per unit area (shear stress) of case A** (when the cilia are perpendicular to the tube wall:Fig. 4a-A) **and case B **(when the cilia bends from the tube wall: Fig. 4a-B) **is the same, how much flow rate is needed for case B?**

The difference in flow rate was estimated when the value of the drag per unit area was the same between (A) when the cilia are perpendicular to the tube wall and (B) when the cilia bend from the tube wall.

When the fluid velocity is written as Eq. (6), the flow rate $Q$ in the circular tubule is written as

17 $$Q=2{\int }_{0}^\frac{R}{2}V\left(r\right)\pi rdr=\cdots =-\frac{\pi }{128\mu }\frac{dp}{dx}{R}^{4}$$

From Eqs. (6) and (17), the following equation is derived:

18 $$V\left(r\right)=\frac{32Q}{\pi {R}^{4}}{r}^{2}$$

Because Eq. (19) is derived from Eqs. (7), (8), and (18), Eq. (20) is finally derived as follows:

19 $$D\left(r\right)=\frac{384\mu aQ}{\pi L{R}^{4}}{r}^{2}dr$$

20 $$D={\int }_{0}^{l}\frac{384\mu aQ}{\pi L{R}^{4}}{r}^{2}dr=\cdots =\frac{128\mu aQ}{\pi L{R}^{4}}{l}^{3}$$

Because the distance ${r}_{c}$ from the tubular wall when the primary cilia are bent in a quarter of an arc can be written as Eq. (21), the drag ${D}_{c}$ applied to the arc-shaped primary cilia is expressed as Eq. (22).

21 $${r}_{c}=\frac{2}{\pi }l$$

22 $${D}_{c}=\frac{128\mu aQ}{\pi L{R}^{4}}{\left(\frac{2}{\pi }\right)}^{3}{l}^{3}$$

Therefore, the drag ${D}_{su}$ per unit area when the primary cilia are perpendicular to the tubular wall and the drag ${D}_{cu}$ per unit area when the primary cilia are bent in an arc can be written as follows:

23 $$D_{su} = {\raise0.7ex\hbox{$D$} \!\mathord{\left/ {\vphantom {D {al}}}\right.\kern-0pt} \!\lower0.7ex\hbox{${al}$}} = \frac{128\mu Q}{{\pi LR^{4} }}l^{2}$$

24 $$D_{cu} = D_{c} /al = \frac{128\mu Q}{{\pi LR^{4} }}\left( {\frac{2}{\pi }} \right)^{3} l^{2}$$

In both cases, assuming that the parameter values ​​other than the flow rate are the same, if ${D}_{su}={D}_{cu}$, the following relationship is derived from Eqs. (23) and (24):

25 $${Q}_{c}={\left(\frac{\pi }{2}\right)}^{3}{Q}_{s}\sim 3.88{Q}_{s}$$

where ${Q}_{s}$ and ${Q}_{c}$ are the flow rates in the case of straight and bent cilia, respectively.

To more accurately represent the cilia, a value based on experimental data^29^ for the cylindrical shape was adopted as ${C}_{D}$.

From the experimental data^29^ of the drag coefficient of the cylinder, for $Re<2$, ${C}_{D}$ can be written as:

26 $${C}_{D}\sim \frac{59}{{Re}^{0.7}}$$

From Eqs. (2), (3), and (26), the following equation is derived:

27 $$D=\frac{59}{2}{\rho }^{0.3}{V}^{1.3}S{\left(\frac{\mu }{L}\right)}^{0.7}$$

Then $D\left(r\right)$ is written as:

28 $$D\left(r\right)=\frac{59}{2}{\rho }^{0.3}a{\left(\frac{\mu }{L}\right)}^{0.7}{V\left(r\right)}^{1.3}dr$$

Using Eqs. (6) and (7), $D\left(r\right)$ can be written as:

29 $$D\left(r\right)=\frac{59}{2\times {4}^{1.3}}{\rho }^{0.3}a\frac{{\alpha }^{1.3}}{{L}^{0.7}{\mu }^{0.6}}{r}^{2.6}dr$$

In addition, if Eq. (18) is used instead of Eq. (6), $D\left(r\right)$ can be written as:

30 $$D\left(r\right)=\frac{59\times {32}^{1.3}}{2\times {\pi }^{1.3}}{\rho }^{0.3}a{{\left(\frac{\mu }{L}\right)}^{0.7}\frac{{Q}^{1.3}}{{R}^{5.2}}r}^{2.6}dr$$

Therefore, the force $D$ can be written as follows:

31 $$D={\int }_{0}^{l}\frac{59\times {32}^{1.3}}{2\times {\pi }^{1.3}}{\rho }^{0.3}a{\left(\frac{\mu }{L}\right)}^{0.7}\frac{1}{{R}^{5.2}}{Q}^{1.3}{r}^{2.6}dr=\frac{59\times {32}^{1.3}}{2\times {\pi }^{1.3}}{\rho }^{0.3}a{\left(\frac{\mu }{L}\right)}^{0.7}\frac{1}{{R}^{5.2}}\frac{1}{3.6}{Q}^{1.3}{l}^{3.6}$$

Then, considering the above discussion, ${D}_{su}$ and ${D}_{cu}$ are written as follows:

32 $$D_{su} = D/al = \frac{{59 \times 32^{1.3} }}{{2 \times \pi^{1.3} }}\rho^{0.3} a\left( {\frac{\mu }{L}} \right)^{0.7} \frac{1}{{R^{5.2} }}\frac{1}{3.6a}Q^{1.3} l^{2.6}$$

33 $$D_{cu} = D_{c} /al = \frac{{59 \times 32^{1.3} }}{{2 \times \pi^{1.3} }}\rho^{0.3} a\left( {\frac{\mu }{L}} \right)^{0.7} \frac{1}{{R^{5.2} }}\frac{1}{3.6a}\left( {\frac{2}{\pi }} \right)^{3.6} Q^{1.3} l^{2.6}$$

In both cases, assuming that all parameter values ​​other than flow rate are the same, if ${D}_{su}={D}_{cu}$, the following relationship is derived from Eqs. (32) and (33):

34 $${Q}_{c}={\left\{{\left(\frac{\pi }{2}\right)}^{3.6}\right\}}^{\frac{1}{1.3}}{Q}_{s}={\left(\frac{\pi }{2}\right)}^\frac{36}{13}{Q}_{s}\sim 3.49{Q}_{s}$$

Comparing Eqs. (25) and (34), it can be seen that the ratio of ${Q}_{c}$ to ${Q}_{s}$ is approximately 10% smaller when experimental data^29^ for cylindrical shapes are used as ${C}_{D}$. Mathematical modeling suggests that increased ciliary curvature reduces the magnitude of drag forces acting on primary cilia under comparable flow conditions. In the case of short and straight cilia, the drag force per unit length was highest at the ciliary tip (Fig. 4b), whereas in the case of long and bent cilia, the peak drag force was shifted toward the midsection (Fig. 4c). This shift in force distribution suggests that, in the cystic model, bending moments generated by fluid flow are more broadly dispersed along the ciliary shaft, potentially reducing the shear stress transmitted to the basal mechanosensitive PC1/PC2 complexes (Fig. 4c). Furthermore, as shown in Eqs 34 of our mathematical model, a urine flow rate approximately 3.49 times higher than that in the model of short and straight cilia would be required for the model of long and bent cilia to experience the same magnitude of drag force.

### Morphological changes of primary cilia in the CKM under higher water intake conditions.

To increase urine output in vivo, 5% glucose was added to the drinking water of CKM rats (high-water intake, HWI). Previous work by Nagao et al. (ref. 30) demonstrated that this intervention leads to a marked increase in urine production, indicating enhanced urinary flow at the whole-kidney level. Although urine flow was not measured at the level of individual tubules, an increase in tubular flow rates would be expected to accompany the observed increase in total urine output. Based on our mathematical model, long and bent primary cilia, as observed in CKM rats under control conditions, are predicted to require an increased flow rate to experience effective mechanical loading comparable to that acting on short and straight cilia. In this context, we analyzed tissue samples from previous HWI studies in which cyst area was reduced and renal function was improved ^30^.

**Fig. 5:**
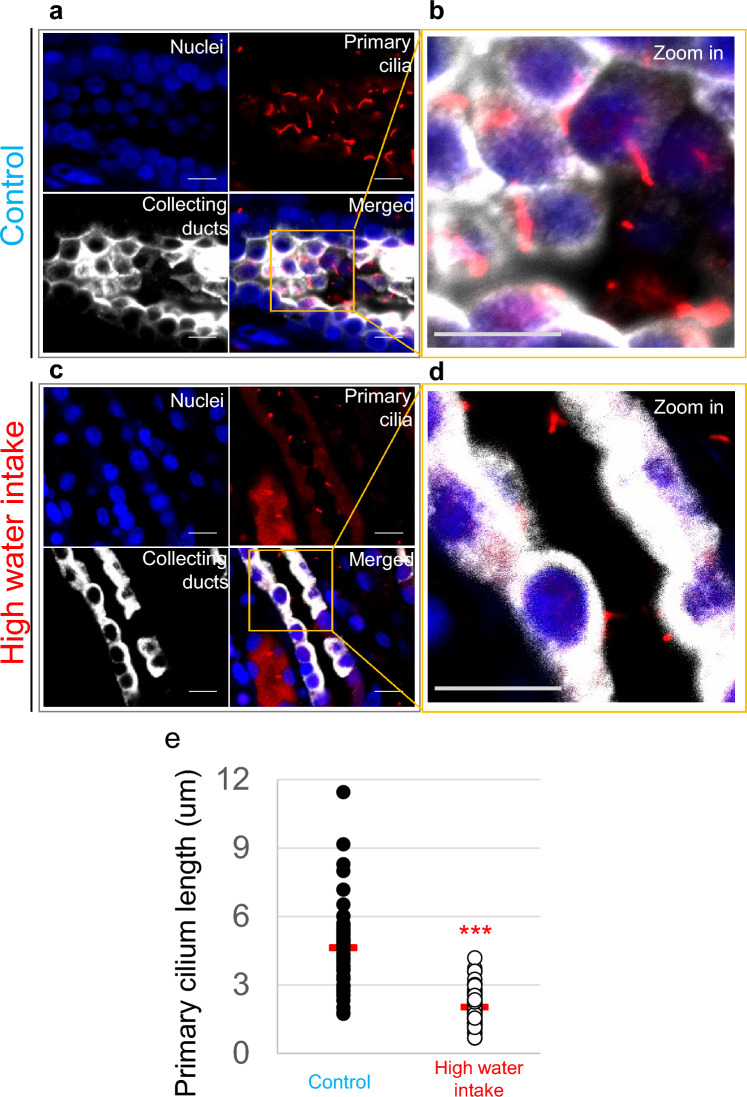
**Morphological changes of primary cilia in CKM under control and high water intake (HWI) conditions.** Wide-field images of collecting ducts from CKM rats under non-treated control conditions (**a**,** b**) and following treatment with HWI (5% glucose with high water intake) (**c**,** d**). Panels c and d represent CKM kidneys exposed to increased urinary flow induced by high water intake (HWI), a condition under which physiologically relevant hydrodynamic loading on primary cilia is predicted by the mathematical model. Panels a and b represent CKM kidneys under baseline conditions, in which the model predicts reduced mechanical loading on elongated and curved primary cilia. Quantitative analysis of primary cilia length is shown in panel e, comparing CKMs exposed to 5% glucose. Primary cilium length represents the calculated straight-line distance (chord length) between the ciliary base and tip derived from orthogonal-axis measurements in Fiji.In Fig. 5, the number of animals is N = 3 for Control and N = 3 for HWI. The number of analyzed cilia is n = 62 for Control and n = 64 for HWI. Each dot represents one cilium; the red bar indicates the mean, and data are presented as mean ± SD in the Supplementary Data. Statistical significance is indicated as ***p < 0.001 (unpaired t-test).

Quantitative analysis revealed that primary cilia in the HWI-treated group were approximately 50% shorter than those in the untreated controls (Fig. 5e). Tubular morphology further showed reduced epithelial overlap and decreased cystic dilation in the HWI group compared with controls (Fig. 5a–d). These morphological changes were observed under conditions in which increased urine output has been reported, consistent with the range of flow enhancement predicted by the model.

## Discussion

Primary cilia function as mechanosensors in renal tubules, and their morphology critically influences the magnitude of mechanical forces generated by tubular fluid flow. In this study, we combined quantitative morphological analysis with a mathematical model to examine how ciliary length and curvature affect flow-induced mechanical loading. Our model predicts that long and curved primary cilia, as observed in cystic kidney models (CKM), experience substantially reduced drag forces, requiring an approximately ≥ 3.49-fold increase in urine flow to achieve mechanical loading comparable to that acting on short, straight cilia in the normal kidney.

Under baseline conditions, urine flow in CKM is therefore expected to remain below this predicted threshold. In contrast, increased water intake has been reported to markedly elevate urine output at the whole-kidney level. Notably, Nagao et al. reported an approximately 7.73-fold increase in urine output under high water intake (HWI) conditions. This magnitude exceeds the model-predicted threshold, placing HWI within the effective range required to restore physiologically relevant mechanical loading on primary cilia. Consistent with this prediction, HWI was associated with shortening and partial straightening of primary cilia, accompanied by improvement of tubular morphology.

The collecting ducts of cystic kidney disease models are the primary sites of cyst formation. During the early stages of cystogenesis, the primary cilia of collecting duct cells sense urine flow and regulate ductal diameter. Under physiological conditions, primary cilia are relatively rigid, toggle-switch-like organelles with axonemes composed of 9 + 0 microtubule doublets. In the CKM, primary cilia were observed to be elongated and bent, with axonemes in which microtubule doublets transition to singlets over approximately half of their total length. Because primary cilia act as sensors that convert physical forces into biochemical signals, alterations in their morphology and length—both of which influence the efficiency of force reception— are expected to reduce their mechanosensory efficiency and potentially contribute to cyst formation.

We present a mathematical model that describes the biomechanical behavior of these long, curved primary cilia. Morphological analysis revealed that CKM cilia are abnormally elongated and curved. Morphological analysis indicated that elongation primarily occurs in the distal segment (DS), which is structurally weaker than the proximal segment (PS) because it contains fewer axonemal microtubules and lower levels of tubulin post-translational modifications that enhance structural stability, as reported in previous studies^31–33^. Consistent with these findings, our SBF-SEM data also demonstrated elongated and curved primary cilia in cystic kidneys. These experimentally observed structural features motivated us to quantitatively examine how ciliary elongation and curvature alter hydrodynamic force transmission under physiological flow conditions. These morphological alterations are predicted to substantially decrease the shear forces acting on the ciliary surface, reducing the bending moment at the ciliary base. To quantify these biomechanical effects, we developed a mathematical model simulating the drag force and bending moment generated by luminal urine flow on elongated, curved primary cilia. The bending moment (torque) at the ciliary base scales with the product of the drag force and the cilium length. Because drag force is proportional to the projected cross-sectional area exposed to flow, ciliary curvature reduces the effective surface area and further decreases the hydrodynamic force. Because drag force encompasses shear-related mechanical components, a reduction in drag may attenuate mechanical stimuli reaching the mechanosensitive PC1/PC2 complexes in primary cilia. Moreover, decreased stiffness and increased curvature may redistribute drag-induced bending moments along the ciliary shaft, potentially reducing the concentration of mechanical stress at the ciliary base, where mechanosensory signaling is thought to be initiated.

Previous studies have suggested that a shear stress threshold of approximately 0.1–1 Pa is required to activate the PC1/PC2 mechanosensitive complex^34–36^. Furthermore, stress concentration at the base of the primary cilium—caused by bending moments and torsional forces—appears to play a critical role in initiating calcium signaling^10,37,38,39^. Therefore, a reduction in effective shear stress—resulting from increased ciliary length, curvature, and decreased stiffness—may impair PC1/PC2 activation. This mechanosensory deficit is likely to suppress intracellular calcium signaling, a key pathway in epithelial homeostasis. Consequently, dysregulated calcium signaling may promote cyst initiation and progression^34,39^.

In summary, our findings suggest that morphological abnormalities of primary cilia in CKM—specifically elongation, curvature, and reduced stiffness—are not merely incidental features but critical determinants of impaired mechanosensation. Quantitative evaluation of these structural and mechanical parameters provides novel insights into CKM pathophysiology and may help identify therapeutic targets aimed at restoring ciliary function.

Our mathematical model predicts that the drag forces acting on elongated and curved primary cilia are reduced to approximately one-quarter of those experienced by straight, short cilia. This substantial reduction corresponds to a significant decrease in shear stress at the ciliary basal body, implying that proportionally greater urine flow is required to restore physiologically relevant levels of mechanotransduction. Accordingly, the model predicts that primary cilia in CKM would require approximately 3.49-fold increase in urine flow rate to experience the same magnitude of drag force as wild-type cilia. These findings suggest that, for proper tissue-level mechanosensory signaling and normalization, the primary cilium would need to be exposed to mechanical loading comparable to that in the normal kidney model.

Taken together, these results indicate that the reduced mechanical stimulation caused by ciliary elongation and curvature in CKM may be partially compensated by increasing urine flow, thereby restoring drag force and shear stress toward physiologically relevant levels.

One approach to increasing urine flow is through water intake. Increased water intake reduces arginine vasopressin (AVP) levels and has been proposed as a simple method to slow renal cyst growth^40–43^. However, therapeutic effects in preclinical CKM models have been inconsistent^28,41,44^. In a previous human clinical trial, a long-term (3-year) adjustment of water intake, indexed by urine osmolality, resulted in a 1.27-fold increase in urine volume^45^ but no reduction in cyst volume, suggesting that the amount of water consumed may not have been sufficient^45,47–52^^.^ This observation aligns with our mathematical model. In an animal model with insufficient urine flow during HWI treatment, Sagar P.S. et al. analyzed its impact on WT (Lewis rats) and CKM (LPK rats) ^44^. Their results showed that the urine flow rate in CKM rats was 2.89-fold^44^ that of WT rats, slightly lower than the 3.49-fold value predicted by our mathematical model. In addition, a decrease in the cystic area has been observed in the results of HWI by Sagar P.S. et al., ^44^ although there is no recovery of renal function.

Conversely, several studies suggest that HWI increases urine flow rate and slows disease progression, allowing renal tubules to approach normal morphology. Hoops K et al*.*^28^ reported a 3.89-fold increase, whereas Nagao S et al*.*^30,41^observed increases of 6.44-fold and 7.73-fold in urine flow rate with HWI, values that meet or exceed the 3.49-fold prediction of our mathematical model. As a result, a slower pathological progression in renal function and structure was observed. Taken together, these findings support the consistency of our mathematical model with prior HWI treatment results and suggest that it is useful for estimating the fluid intake required to achieve therapeutic efficacy. To further evaluate the effect of applying the same drag force to primary cilia in CKM as in the normal kidney model, as predicted by our mathematical model, we analyzed samples from Nagao et al*.* (2019)^30^ that reported reduced cyst area and improved renal function. The results showed that HWI treatment (weeks 4–20 of age) led to a 0.51-fold reduction in primary cilia length and slower disease progression compared with age-matched CKM rats that did not receive HWI. Primary cilia are known to regulate tubule diameter^53,54^. Primary cilia length positively correlates with cyst area^55^, and genetic manipulation that shortens primary cilia in cystic kidneys has been reported to suppress cyst formation^56,57^. Fluid flow above a certain threshold maintains primary cilia at a shorter length through activation of the mechanosensitive PC1/PC2 complex^58–60^. In contrast, cilia elongate under insufficient flow^61^. This flow-dependent length regulation has been observed in multiple in vitro models and confirmed in vivo^58,59,61,62^, indicating that cilia of appropriate length reflect exposure to appropriate mechanical stimuli. Therefore, it is intriguing that the primary cilia are shortened and the tubules are normalized by the HWI treatment, which gives the same drag force as in normal kidney model.

In normal kidneys, the length of primary cilia is regulated by the flow rate of urine^63^. In our mathematical model, primary cilia in the CKM are elongated and curved, leading to a significant reduction in the drag force per unit surface area (shear stress) exerted by the urine flow. This indicates that increasing urine flow to levels predicted by the model may help restore mechanosensory function. Given that primary cilia play a crucial role in sensing fluid flow and modulating tubular architecture, such an increase in flow could help normalize tubule morphology. Therefore, the mathematical model presented here may serve as a predictive tool for estimating the minimum water intake required to restore physiological drag forces equivalent to those experienced by primary cilia in healthy kidneys, potentially providing a quantitative basis for optimizing hydration-based therapeutic strategies for cystic kidney disease. Moreover, because high-water intake is not always feasible due to patient discomfort and the risk of water intoxication, this mathematical model may provide a framework for identifying or optimizing alternative therapeutic approaches—such as pharmacological agents or mechanical interventions—that can restore appropriate shear stress to primary cilia.

Finally, we note that the inferred differences in hydrodynamic loading are based on physiological surrogates (urine output) rather than direct flow measurements, positioning our estimates as relative rather than absolute values. Accordingly, although our model captures how aberrant ciliary geometry redistributes hydrodynamic forces, several limitations should be acknowledged. Primary cilia can display local diameter enlargements such as ciliary bulbs, which would proportionally elevate the drag acting on those segments^64–67^. In our formulation, such effects could be incorporated by locally replacing the radius term with an increased value when evaluating segment-wise drag and during spatial integration; however, bulbs were not explicitly modeled here. While tortuosity analysis revealed a population-level shift toward increased bending in CKM, tortuosity alone does not uniquely specify three-dimensional morphology. The quarter-circle configuration used here therefore represents an idealized reference shape chosen for analytical tractability rather than an attempt to reconstruct specimen-specific geometries.

Our framework also adopts a “deformation → force redistribution” perspective, in which experimentally observed shapes are treated as given and used to compute drag and torque under laminar flow. This static abstraction facilitates mechanistic interpretation but does not include local elastic deformation, stochastic curvature, segment-specific flow, or time-dependent remodeling that would emerge in fully dynamic fluid–structure interaction models. Similarly, because segment-specific tubular flow was not available, whole-kidney urine output was used as a physiological surrogate to scale hydrodynamic loading; direct measurements of luminal flow would refine quantitative predictions. In addition, minor differences in absolute ciliary length values across figures reflect methodological variability inherent to projection-based chord-length measurements—such as Z-sampling density, axial resolution, and manual base–tip identification—rather than biological discrepancies, as supported by the comparable distribution shapes observed across analyses.

Moreover, hydrodynamic loading represents only one component of high-water-intake biology. Flow-independent factors—such as altered osmolarity or electrolyte composition, epithelial tension, metabolic or inflammatory states, or vasopressin/cAMP-dependent regulation of ciliary length—may interact with or potentiate the mechanical effects described here. Finally, although CKM lacks Pkd1/2 mutations, disease-associated epithelial changes and abnormal ciliary geometry may distort force transmission to the polycystin complex, potentially impairing mechanotransduction through aberrant mechanical input rather than intrinsic channel dysfunction. Future integrative models linking geometry, elasticity, biochemical signaling, and hormonal regulation will be required to fully resolve how ciliary structure governs mechano-sensation in cystic kidney disease. This addition reinforces the value of our simplified model while explicitly outlining its natural extensions for more detailed future implementations.

## Material and methods

### Animals

The PCK rat strain (CKM: cystic kidney model) was originally derived from a Sprague–Dawley colony at Charles River, Tokyo, Japan. This strain, characterized by a splicing mutation with subsequent skipping of exon 36 and a frameshift in the human orthologous *Pkhd1* gene, leads to renal cysts originating from the collecting ducts and congenital hepatic fibrosis complicated by biliary cysts^30,41^. Sprague–Dawley males, also obtained from Charles River, Japan, were used as the normal kidney model (WT: wild-type) animals. Rats were bred and maintained at the Advanced Medical Research for Animal Models of Human Diseases at Fujita Health University. All animals used in this study were provided ad libitum access to laboratory food and water. Animals were anesthetized with 3–5% isoflurane using an induction chamber for 5 min and maintained with a nose cone for continuous delivery of 3% isoflurane. Depth of anesthesia was confirmed by the absence of pedal reflex. A midline abdominal incision was made, and blood was collected from the heart, resulting in euthanasia by exsanguination under deep anesthesia, confirmed by cessation of heartbeat and respiration, in accordance with institutional guidelines. Animal studies are reported in compliance with the ARRIVE guidelines.

### HWI treatment

To increase the urine flow rate in vivo, 5% glucose was added to water for the CKM. This method (HWI: high-water intake) was reported in Nagao et al*.*, (2019)^30^. In short, PCK rats and normal Sprague–Dawley (+ / + ;WT) rats were allowed free access to water and food throughout the study. PCK and + / + rats were randomly assigned to either the control group (normal tap water) or the HWI group (water that contained 5% glucose) and treated as such from 4 to 20 weeks of age. Urine volume per unit time was used as a physiological surrogate for tubular flow (Qs) in the model, serving as a relative scaling parameter rather than a direct measurement of flow in individual nephrons or collecting ducts. Because the dataset does not include direct measurements of tubular flow, Qc was estimated from urine output, providing a physiologically grounded but indirect proxy for hydrodynamic load.

Hydrodynamic loading was inferred rather than directly measured. Following Nagao et al., we defined the 5% glucose–supplemented group as HWI and unsupplemented controls as Cont. Because single-tubule flow values were unavailable, urine output was used as a physiological surrogate for Qc, leveraging the established glucose-induced increase in water intake and polyuria. The model therefore evaluates relative—not absolute—flow differences between groups.

Because HWI treatment results in an increase in urine flow, it was hypothesized that experimental urine flow would match or surpass the theoretical values compared with our mathematical model. As a result, the HWI therapy reported in 2019^30^ exceeded the theoretical value of our mathematical model. Therefore, we observed the morphology of primary cilia and renal tubules. Kidneys from HWI-treated CKM (PCK) and WT rats were also used for observation of cilia length and morphological analysis of renal tubule.

### TEM and data analysis

Samples for EM were fixed using 4% PFA + 2.5% GA in PBS and then embedded in Quetol 812 epoxy resin (Nissin EM, Tokyo, Japan).

Ultrathin sections were cut to 70 nm – 80 nm thickness with a diamond knife on an ultramicrotome, mounted on a copper grid, and double stained with uranyl acetate and lead citrate. Finally, they were observed with a transmission electron microscope (JEM-1400 flash; JOEL, Tokyo, Japan) at an acceleration voltage of 80 kV.

### SBF-SEM and data analysis

For SBF-SEM, the tissue was fixed using 4% PFA and 1% glutaraldehyde dissolved in 0.1 M PB. The tissue was post-fixed and stained en bloc, using 2% OsO4 containing 1.5% K4[Fe(CN)6], 1% thiocarbohydrazide, 2% OsO4, uranyl acetate, and lead aspartate^68^. Thereafter, the sample was dehydrated and embedded in Durcupan (Sigma-aldrich) with 5% carbon black added to increase the conductivity of the resin^69^. Blocks from each group were trimmed and attached to aluminum rivets using conductive adhesive (Chemtronics, Kennesaw, GA, USA). The surfaces of the trimmed specimens were sputtered with gold to enhance conductivity and mounted on a MERLIN or SIGMA/VP SEM system (Carl Zeiss, Jena, CA, USA) equipped with a 3-View in-chamber ultramicrotome (Gatan, Pleasanton, CA, USA), and then the samples were observed and captured. Serial electron microscopic images were processed with Fiji/ImageJ (http://fiji.sc/wiki/index.php/Fiji) and segmented with Microscopy Image Browser (http://mib.helsinki.fi/)^70^. The segmented data were reconstructed into 3D images with Amira (Thermo Fisher Science). For 3D electron microscopy analyses, collecting duct samples were obtained from two CKM rats. A total of three collecting duct segments were reconstructed in three dimensions, reflecting the practical constraints of the limited field of view inherent to high-resolution 3D-EM. Each reconstructed dataset contained multiple epithelial cells and their associated primary cilia. Measurements were performed at the individual cilium level and pooled for statistical analysis, while the number of source animals was explicitly indicated to distinguish biological replication from technical sampling. Despite this sampling structure, consistent tendency of morphological features were observed across specimens, including increased mean ciliary length and higher tortuosity in the CKM model compared with WT.

### Quantification of primary cilium length

Primary cilium length was quantified using two definitions depending on the analysis. For three-dimensional reconstructions (Fig. 2), cilia were traced along their curved centerline to obtain arc-length measurements. Straight-line distances between the ciliary base and tip (chord length) were also extracted from the same 3D datasets. In Figs. 3 and 5, cilium length was defined as a calculated chord length obtained from manual measurements along orthogonal axes in Fiji, where the base, tip, and the tip’s projection onto the xy-plane form a right triangle whose hypotenuse corresponds to the cilium length.

### Cilium morphology analysis

Cilium length and curvature were quantified from reconstructed traces using Amira. The tortuosity of each cilium was calculated as the ratio of its curved length (a) to the chord length (b) connecting the base and tip:

$${\mathrm{Tortuosity}} = \, \left( {\mathrm{a}} \right)/\left( {\mathrm{b}} \right)$$

A tortuosity value of 1 corresponds to a perfectly straight cilium, whereas higher values indicate increased curvature or convolution (Supplemental Information Data). This measurement corresponds to the schematic shown in Fig. 1.

### Immunofluorescence microscopy

Immunofluorescence microscopy was performed using an LSM710 confocal microscope (LSM710; Carl Zeiss) with an Axiovert 200 M microscope (Carl Zeiss), a Plan Apochromat 63 × /1.4 NA oil immersion lens, and ZEN software (Carl Zeiss), as described previously^71,72^. The procedure involved fixing WT and PCK rat kidneys in 4% paraformaldehyde for two nights, embedding them in paraffin, and processing deparaffinized sections in 10 mM sodium citrate buffer (pH 6.0). Sections were then blocked with 5% (w/v) BSA in PBS and processed for immunofluorescence (incubation at 4 ℃ overnight with the 1st antibody included 1:400 monoclonal anti-acetylated tubulin [T7451, sigma-aldrich], 1:500 anti-c-terminal region of aquaporin2 [OSA00145, Osenses Pty Ltd.], and 1:300 biotinylated anti-polyglutamylation modification mAb [GT335, polyE] antibodies). This was followed by a 2-h at 26 ℃ incubation with the 2nd antibody: Alexafluor 488, 555, or 647 conjugated streptavidin, anti-mouse, or rabbit IgG donkey antibody.

Z-stacked images (0.1-um pitch) were used to determine the length of the cilium and the structure of the ciliary axoneme. Cilia length was calculated by considering the points corresponding to the ciliary base and tip forming a right-angle triangle, with a third point corresponding to a projection of the tip on the x and y planes. In this triangle, the hypotenuse was the cilia length. To calculate cilia length, points corresponding to the ciliary base and tip form a right triangle with a third point corresponding to a projection of tip on the x, y plane. In this triangle, the hypotenuse is the cilia length. All images were taken at room temperature. The ciliary length was measured using Fiji software (National Institutes of Health).

### Statistics

Statistical analyses were performed using individual cilia as the unit of analysis (n), while the number of animals (N) in each group is reported to indicate biological replication. For Fig. 2, WT and CKM groups included N = 3 datasets from 2 animals each, with n = 27 and n = 25 analyzed cilia, respectively. For Fig. 3, both WT and CKM groups had N = 3 animals, with n = 18 and n = 48 cilia. For Fig. 5, control and treatment CKM groups each consisted of N = 3 animals, with n = 62 and n = 64 cilia. Data distribution was assessed for normality prior to analysis. For two-group comparisons, unpaired two-tailed Student’s t-tests were used for normally distributed data, and Mann–Whitney U tests for non-normal data. For comparisons among multiple groups, one-way ANOVA followed by Tukey’s post hoc test was applied. Data are presented as mean ± SD or as median with interquartile range, as indicated in the figure legends. Exact p-values are provided in the figure legends, and statistical significance was defined as *p* < 0.05. Statistical analyses were performed using GraphPad Prism (Fig. 2h–i) and StatView (Figs. 3 and 5).

## Supplementary Information

Supplementary Information 1.

Supplementary Video 1.

Supplementary Video 2.

## Data Availability

All relevant data supporting the findings of this study are available within this article, its Supplementary Information and Source Data files. Source data are provided with this paper.
